# Endothelium Activation Markers in Severe Hospitalized COVID-19 Patients: Role in Mortality Risk Prediction

**DOI:** 10.1055/s-0041-1731711

**Published:** 2021-07-04

**Authors:** Marina Marchetti, Patricia Gomez-Rosas, Eleonora Sanga, Sara Gamba, Cristina Verzeroli, Laura Russo, Francesco Restuccia, Francesca Schieppati, Ezio Bonanomi, Marco Rizzi, Stefano Fagiuoli, Andrea D'Alessio, Luca Lorini, Anna Falanga

**Affiliations:** 1 Department of Immunohematology and Transfusion Medicine, Hospital Papa Giovanni XXIII, Bergamo, Italy; 2 Hematology Service, Hospital General Regional Tecamac IMSS, Mexico, Mexico; 3 Department of Anesthesiology and Critical Care Medicine, Hospital Papa Giovanni XXIII, Bergamo, Italy; 4 Unit of Infectious Diseases, Hospital Papa Giovanni XXIII, Bergamo, Italy; 5 Department of Internal Medicine, Hospital Papa Giovanni XXIII, Bergamo, Italy; 6 Department of Medical Oncology and Internal Medicine, Policlinico San Marco, Zingonia, Bergamo, Italy; 7 School of Medicine, University of Milano Bicocca, Milano, Italy

**Keywords:** endothelium, COVID-19, von willebrand factor, hypercoagulation

## Abstract

**Introduction** Endothelial damage and hypercoagulability are major players behind the hemostatic derangement of SARS-CoV-2 infection.

**Aim** In this prospective study we assessed endothelial and inflammatory biomarkers in a cohort of COVID-19 patients, aiming to identify predictive factors of in-hospital mortality.

**Methods** COVID-19 patients hospitalized in intensive care (ICU) and non-ICU units at 2 Bergamo (Italy) hospitals from March 23 to May 30, 2020, were enrolled. Markers of endothelium activation including von-Willebrand factor (vWF), soluble thrombomodulin (sTM), and fibrinolytic proteins (t-PA and PAI-1) were measured. Additionally, D-dimer, Fibrinogen, FVIII, nucleosomes, C reactive protein (CRP) and procalcitonin were assessed.

**Results** Sixty-three (45 ICU, and 18 non-ICU) patients, with a median age of 62 years were analyzed. Increased plasma levels of D-dimer, FVIII, fibrinogen, nucleosomes, CRP, and procalcitonin were observed in the whole cohort. Extremely elevated vWF levels characterized all patients (highest values in ICU-subjects). After a median time of 30 days, death occurred in 13 (21%) patients. By multivariable analysis, vWF-activity, neutrophil-count and PaO2/FiO2 were significantly associated with death. Using these variables, a linear score with 3-risk groups was generated that provided a cumulative incidence of death of 0% in the low-, 32% in the intermediate-, and 78% in the high-risk group.

**Conclusions** COVID-19-induced hemostatic abnormalities are exacerbated by the severity of the disease and strongly correlate with the inflammatory status, underlying the link between coagulation, endothelial activation, and inflammation. Our study provides evidence for a role of vWF, together with neutrophils and PaO2/FiO2, as a significant predictor of in-hospital mortality by SARSCoV-2 infection.

## Introduction

Patients with severe COVID-19 pneumonia are characterized by a hypercoagulable state and high incidence of thrombotic events, affecting both the venous and arterial districts. 1 2 3 4 SARS-CoV-2, the virus responsible of COVID-19, primarily affects the lungs, causing interstitial pneumonitis and acute respiratory distress syndrome (ARDS), however other organs, particularly the cardiovascular system, are also affected. 5 Several studies till date have suggested a dysregulated host immune response as the major cause of COVID-19-induced mortality, 6 7 however a key role of the endothelium, particularly the pulmonary one, is also highlighted. 8 9

Histopathological studies have indeed shown that an endothelial dysfunction occurred in severe COVID-19 patients, 10 11 12 a condition that, together with a generalized inflammatory state, can significantly contribute to the onset the observed disease-associated coagulopathy. 13 Evidence suggests that endothelial dysfunction in COVID-19 is the result of several indirect and direct viral-dependent mechanisms acting on endothelial cells. 8 Specifically, indirect mechanisms of endothelial perturbation are related to the effect of proinflammatory effects of cytokines and angiogenic factors excessively released by immune cells and acting on vascular endothelium. 14 Differently, direct mechanisms are linked to the capacity of SARS-CoV-2 to infect endothelial cells through the angiotensin-converting enzyme 2 (ACE2) expressed on their cell membrane, 15 16 to proliferate within the cells and consequently to induce cell damage and apoptosis. 12

Once activated, endothelium reverts its anti-thrombotic phenotype to a prothrombotic one, 17 characterized by increased expression of cell surface adhesion molecules (i.e., ICAM-1, E-selectin, P-selectin, and VCAM-1) 18 ; downregulation of anticoagulant properties (i.e., thrombomodulin [TM], tissue factor pathway inhibitor [TFPI], and heparan sulfate) 19 ; increased expression of procoagulant (Tissue Factor, TF) and antifibrinolytic activities (plasminogen activator inhibitor 1, PAI-1). 20 The upregulation of TF and PAI-1, and down-regulation of TM and TFPI, promote hypercoagulation and excessive fibrin formation. 21 22 In addition, activated endothelium produces and releases additional cytokines that stimulate the recruitment of inflammatory cells, including neutrophils and platelets, promoting vascular inflammation and permeability. 23 Finally, the acute release of unusually large von Willebrand factor (vWF) multimers from storage granules of activated endothelium favors platelet adhesion and microthrombi formation. 24 Therefore, a vasculopathy can coordinate the destructive microvascular coagulopathy associated with SARS-CoV-2 infection, as also demonstrated by the association of circulating markers of endothelial perturbation with critical illness and death as well as with prothrombotic manifestations. 6 25 These findings support the known tight connection between thrombosis and inflammation, 13 26 two processes mutually reinforcing each other. In this study, conducted in two-centers at the peak of the pandemic in Bergamo, Italy, in a prospective cohort of consecutive COVID-19 patients hospitalized in ICU and non-ICU-wards, we evaluated a series of endothelial activation biomarkers together with biomarkers of coagulation activation and inflammation in relation to disease severity and ARDS. Final goals were to identify laboratory and clinical values potentially predictive of in-hospital mortality and to develop a predictive risk assessment model.

## Methods

### Study Subjects and Patient Classification

Adult patients (≥18 years old) with a confirmed diagnosis of COVID-19 pneumonia and hospitalized at the Hospital Papa Giovanni XXIII and at the Policlinico San Marco, Gruppo San Donato of Bergamo between March 23 ^rd^ to May 1 ^st^ , 2020, were enrolled into the EMOCOVID study (registered at clinicaltrials.gov identifier # 04595110). The study protocol was approved by the local Ethics Committee that waived the need for consent. The study was conducted according to the last revision of the Helsinki Declaration. Patients were admitted in intensive care (ICU) or non-ICU units, according to the disease severity. Patients were enrolled into the study within 2 weeks from hospitalization. The following data were recorded: age, gender, relevant comorbidities: type 2 diabetes, arterial hypertension, atrial fibrillation, active cancer, and others (i.e., dyslipidemia, cardiovascular disease, renal insufficiency, obesity); body mass index (BMI), oxygen therapy. ARDS severity was classified based on the ratio of partial pressure of arterial oxygen and fractional concentration of oxygen inspired air (PaO2/FiO2), according to the current definition of ARDS for oxygenation (PaO2/FiO2 ratio of 300 to 200mmHg: Mild, 200 to 100mmHg: Moderate and less than 100mmHg: Severe ARDS). 27 COVID-treatment was given at the discretion of the treating physicians and the current hospital-protocols for the health emergency and included antivirals, steroids, hydroxychloroquine, and tocilizumab. Anticoagulant therapy (i.e., low molecular weight heparin [LMWH] or unfractionated heparin [UFH]) was provided based on the risk stratification of patients and the International Society of Thrombosis and Hemostasis (ISTH) guidance in SARS-CoV-2, 28 as well as antiaggregant therapy. The primary outcome of this analysis was in-hospital mortality.

Blood samples obtained from a group of 108 (75M/33F) healthy subjects with a median age of 49 years (range: 35–64 years) recruited from hospital employees served as control samples for coagulation testing. Healthy subjects signed a specific informed consent for the use of their blood samples. Subjects were free of cardiovascular disease, thrombotic or bleeding disorders, diabetes, cancer, or infectious diseases, and were not taking anti-platelet, or anti-inflammatory drugs in the last 10 days before blood sampling, nor anti-coagulant drugs.

### Blood Samples

Blood sampling was performed using a 21-gauge needle. After discarding the first 2–3 mL, the blood was collected into sterile siliconized tubes (BD Vacutainer® Blood Collection Tubes, Becton, Dickinson) containing 0.11M Citrated Sodium for coagulation studies, and K3-ethylenediamine tetraacetic acid (K3-EDTA, 7.2 mg) for the complete blood cell count study. White blood cell differential count, hematocrit, hemoglobin, red blood cell count and platelet count were determined by a Sysmex-XE 2100 hematology analyzer (Sysmex, Kobe, Japan). Plasma was obtained by two sequential centrifugations at 2,600xg for 15 minute at 25°C and isolated plasma was aliquoted and stored at -80°C. Plasma samples were thawed “for 5 minute” in a 37°C water bath, homogenized and stored at room temperature for a maximum of 20 minute before starting the tests.

### Coagulation Assays

Fibrinogen was measured by Clauss's method on a STA Compact Max3 analyzer (STA-Liquid Fib), while FVIII coagulant activity (HemosIL FVIII:c, Werfen, Italy) and D-dimer (HemosIL D-dimerHS, Werfen) were measured on an ACL TOP 500 coagulation analyzer (Werfen), according to manufacturer's instructions.

### Endothelial Activation Markers

Plasma levels of von Willebrand factor (vWF) antigen (HemosIL vWF Antigen, IL), and vWF ristocetin-cofactor activity (HemosIL vWF Ricof, IL) were measured on an ACL TOP 500 coagulation analyzer (Werfen group, Italy) according to the manufacturer's instructions. Plasma levels of soluble thrombomodulin (sTM) were determined by commercially available ELISA (Abcam, ab46508 System) and results expressed in ng/ml.

### Fibrinolytic Proteins

Plasminogen activator inhibitor type 1 (PAI-1 Antigen, Zymutest, HYPHEN BioMed), and tissue plasminogen activator (t-PA, Zymutest, HYPHEN BioMed) were determined by commercially available ELISA testing.

### Inflammation and Leukocyte Activation Markers

Upon activation, neutrophils release in circulation granular enzymes and chromatin that together form neutrophil extracellular traps (NETs). Nucleosomes which consist of cleaved DNA/cytoplasmic histone-associated-DNA-fragments were determined in plasma samples as circulating NETosis products using the Death Detection ELISAPLUS kit (ROCHE Diagnostics) according to the manufacturer's instructions. Results are expressed in arbitrary units or AU (1 AU = 0.001 OD). Interleukin 6 (IL 6) plasma levels were assayed by ELISA kit (BioSource/Invitrogen Hu) according to the manufacturer′s instructions. C-reactive protein (CRP) and procalcitonin concentrations were measured by immunoturbidimetric assays on ADVIA 2400 (Siemens Healthcare Diagnostics USA).

### Statistical Analysis

Categorical data are summarized as frequencies and proportions, while continuous variables are summarized as mean and standard deviation or median and 5 ^th^ -95 ^th^ percentile range depending on their distribution. Student's *t* -test, Pearson's Chi2, or Mann-Whitney test was employed to compare the difference between the mean values of different groups. Statistical inference is based on two-sided tests of the null hypothesis considering significance at a p-value < 0.05. Pearson's correlation coefficient and linear regression were used to associate continuous variables. Survival functions were estimated using the Kaplan-Meier method, baseline time is assumed at the time of recovery, while survival analyses were performed using the multivariable linear regression analysis. The predictive variables of the mortality score were identified by multivariable linear regression analysis, by estimating the regression coefficients (i.e., the estimated effect) values, the standard error of the estimate and the p-value. Discrimination ability of the score was assessed by evaluating the area under the receiver operating curve (ROC). The predictive accuracy of the logistic regression model was also assessed by the bootstrap-based optimism correction method. 29 Statistical analysis was performed by using SPSS v26.0 (IBM Corp) and the Prism software version 8 (GraphPad software, Inc).

## Results

### Clinical Characteristics of the Study Population

A cohort of 63 consecutive adult patients with COVID-19 infection admitted to both non-ICU ( *n* = 18; 29%) and ICU ( *n* = 45; 71%) were enrolled in the study. Table 1 shows the demographics and clinical characteristics of the patients at the time of study inclusion. Median age of study cohort was 62 years (range 35–88 years) and most of the patients were male ( *n* = 45; 71%). Oxygen support in the ICU patients was at high-flow in 42 (93%) and low-flow in the remaining 3 (7%) patients, while, in the non-ICU group, 2 patients were under high-flow oxygenation (11%), and 16 (89%) under low-flow. At enrollment, the mean Horowitz oxygenation index, i.e., the PaO2/FiO2 ratio, was 103 mm Hg in the ICU group and 160 mm Hg in the non-ICU patients ( *p* < 0.028). Based on the degree of hypoxemia by PaO2/FiO2 values, 40 (64%) patients were diagnosed with an acute respiratory distress syndrome (ARDS), 6 of them with mild, 17 with moderate and 17 with severe ARDS. COVID-19 inpatient medications consisted of antivirals (non-ICU vs ICU: 28% vs 62%), steroids (61% vs 51%), hydroxychloroquine (44% vs 71%), and tocilizumab (6% vs 24%). Forty-seven patients (74%) received some type of anticoagulant treatment, 20 (32%) with prophylactic dose LMWH and 27 (43%) with therapeutic doses of LMWH or UFH. Moreover, 19 (30%) patients were on antiplatelet therapy.

**Table 1:** Baseline characteristics of the study population according to Non-ICU and ICU hospitalization

	Non-ICU patients ( *N* = 18)	ICU patients ( *N* = 45)	*p*
Male gender (%)	10 (56)	35 (78)	0.178
Age (years [mean/SD])	69 ± 13	62 ± 9	0.052
**Comorbidities (%)**	17 (94)	31 (69)	**0.032**
• Type 2 Diabetes	5 (28)	2 (4)	
• Arterial hypertension	3 (17)	18 (40)	
• Atrial fibrillation	5 (28)	5 (11)	
• Active cancer	2 (10)	4 (9)	
• Other	2 (10)	2(4)	
**PO2/FiO2** (mmHg[median/95%IC])	160 (86–313)	103 (55–226)	**0.028**
**ARDS severity according to PaO2/FiO2 (%)**			
• Mild 200–300 mm Hg	4 (22)	2 (4)	
• Moderate 100–200 mm Hg	3 (17)	14 (31)	
• Severe <100mmHg	2 (11)	15 (33)	
**Routine laboratory parameters** (% out Ref. Value)			
• Leukocytes [Ref. Value: 4.2–9.4*10 ^9^ /L]	7 (39)	35 (78)	0.202
• Neutrophils [Ref. Value:2.0–6.7*10 ^9^ /L]	6 (33)	30 (67)	0.074
• Lymphocytes [Ref. Value: 1.13–3.40*10 ^9^ /L]	8 (44)	30 (67)	0.345
• Hematocrit [Ref. Value: 37.9–46.1%]	9 (50)	38 (84)	**0.018**
• Platelets [Ref. Value: 150–400*10 ^9^ /L]	4 (22)	21 (47)	
• PT ratio [Ref. Value: <1.2]	10 (56)	22 (49)	
• aPTT ratio [Ref. Value: <1.2]	10 (56)	30 (67)	
**COVID-19 treatment (%)**			
• Antivirals	5 (28)	28 (62)	**0.006**
• Steroids	11 (61)	23 (51)	0.760
• Hydroxychloroquine	8 (44)	32 (71)	**0.022**
• Tocilizumab	1 (6)	11 (24)	
**Antithrombotic strategy (%)**	16 (89)	31 (69)	0.678
• ***LMWH***	13 (72)	22 (49)	
▪ Prophylactic dose	8 (44)	5 (11)	
▪ Therapeutic dose	5 (28)	17 (38)	
• ***UFH***	2 (11)	7 (16)	
▪ Prophylactic dose	2 (11)	2 (22)	
▪ Therapeutic dose	−	5 (11)	
*** Fondaparinux***	−	2 (4)	
• ***VKA***	1(6)	−	
• Antiplatelet	2 (11)	17 (38)	

Data are presented as number (percentage). P is statistical significance by Pearson's chi-square test or by Mann- Whitney test. PaO2/FiO2 index is expressed as median, (95%IC). Ref. Value: Reference values; LMWH: low molecular weight heparin, UFH: unfractionated heparin, VKA: vitamin K antagonists. Antiplatelet (Acetylsalicylic acid 100mg/day or Clopidogrel 75mg/day).

As shown in Table 1 , 60% of the total patient cohort had a leukocyte count above the reference value, with clear neutrophilia in 57% and lymphopenia in 60% of them. ICU subjects displayed significantly ( *p* < 0.05) higher total leukocyte and platelet counts compared with non-ICU patients. Total leukocyte (r= -0.601) and neutrophil (r= -0.622) counts were inversely correlated with PaO2/FiO2 ratio values ( *p* < 0.001).

### Coagulation Profile and Hypercoagulability

**Fig. 1:**
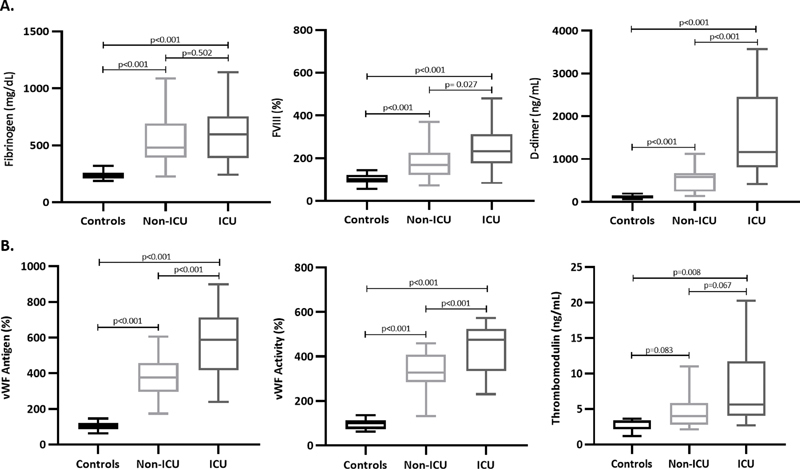
Hypercoagulability (panel A) and endothelial (panel B) biomarkers in non-ICU and ICU patients compared with controls.

PT and aPTT were prolonged in 51% and 64% of total patients, respectively ( Table 1 ). aPTT prolongation was associated with administration of therapeutic doses of UFH (r= 0.250; *p* = 0.05). Hyperfibrinogenemia (i.e., fibrinogen > 400 mg/dL) was observed in 75% of the patients together with significant rise in FVIII activity ( *p* < 0.001), with the highest levels in the ICU group. Finally, increased D-dimer levels were detected in the overall cohort of patients, with ICU patients displaying significantly higher mean values as compared with non-ICU subjects ( Fig. 1A ). We applied the ISTH score 30 for compensated and non- compensated disseminated intravascular coagulation (DIC) in the whole group of patients, as well as in the sub cohort of ICU and non-ICU patients ( Table 2 ). By ISTH score, only 5 patients from the total cohort (4 from the ICU and 1 from non-ICU group) achieved the criteria for overt DIC, interestingly these patients did not show any prolongation of aPTT and PT assays.

**Table 2:** ISTH score for compensated and non-compensated DIC in the whole group of patients, as well as in the cohort of ICU and non-ICU patients

ISTH score	All patients N= 63 (%)	ICU N= 45 (%)	Non-ICU N= 18 (%)
0	3 (5)	0 (0)	3 (17)
1	9 (15)	6 (13)	3 (17)
2	7 (11)	3 (7)	4 (22)
3	12 (19)	8 (18)	4 (22)
4	26 (42)	24 (53)	2 (11)
5	3 (5)	3 (7)	0 (0)
6	2 (3)	1 (2)	1 (6)

Abbreviations: DIC, disseminated intravascular coagulation; ISTH, International Society of Thrombosis and Hemostasis.

Data are presented as number (percentage).

### Endothelium Activation Biomarker Study

#### Von Willebrand Factor

**Fig. 2:**
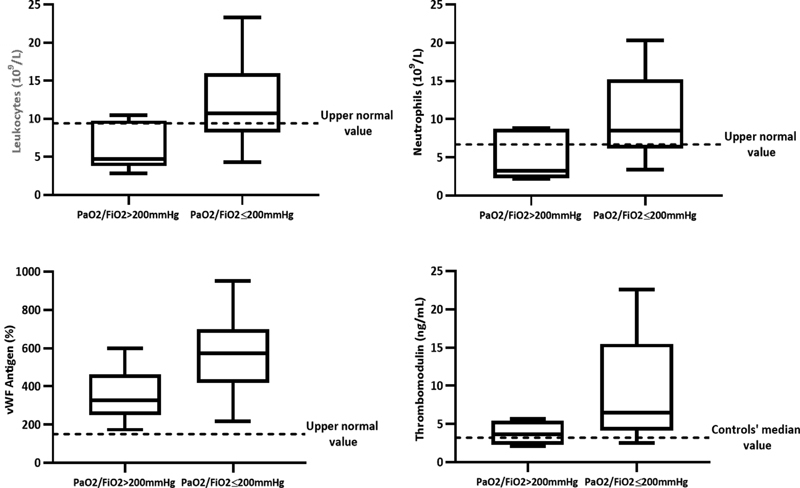
Inflammatory and endothelial biomarkers in relation to low (PaO2/FiO2 >200mmHg) and moderate-severe ARDS (PaO2/FiO2 ≥200mmHg).

vWF levels, measured as both antigen and activity, were significantly greater in the entire group of patients, with all subjects presenting values higher than the upper normal reference limit (i.e., 150%). According to disease severity, ICU patients displayed higher median vWF antigen and activity levels compared with non-ICU patients ( Fig. 1B ). Significant associations were found between vWF antigen and vWF activity (r= 0.928; *p* < 0.001), FVIII (r= 0.480; *p* < 0.001) and D-dimer (r= 0.352; *p* = 0.008), also after multivariable analysis corrected for sex and gender. Additionally, vWF antigen (r= -0.445; *p* = 0.004) and activity (r= -0.457; *p* = 0.003) levels were inversely associated with PaO2/FiO2 values. After multivariable regression analysis including age and gender, high vWF levels were still associated with low PO2/FiO2 (B= -0.450; *p* = 0.005). Specifically, we could observe that patients with moderate to severe ARDS (i.e., with values of PaO2/FiO2 ≤ 200%) were characterized by significantly ( *p* < 0.05) higher levels of vWF antigen, leukocyte, and neutrophil counts, compared with patients with mild ARDS (i.e., PaO2/FiO2 >200%) ( Fig. 2 ).

#### Soluble Thrombomodulin

The overall group of patients displayed significantly ( *p* = 0.004) higher circulating sTM levels than controls, without statistically significant differences between non-ICU and ICU subjects ( Fig. 1B ). However, data analyzed according to ARDS severity, showed that patients with a moderate to severe ARDS (i.e., PaO2/FiO2 ≤ 200%) displayed significantly ( *p* < 0.028) higher sTM levels compared with patients with mild ARDS (i.e., PaO2/FiO2 > 200%) ( Fig. 2 ).

### Fibrinolytic Protein Study

**Fig. 3:**
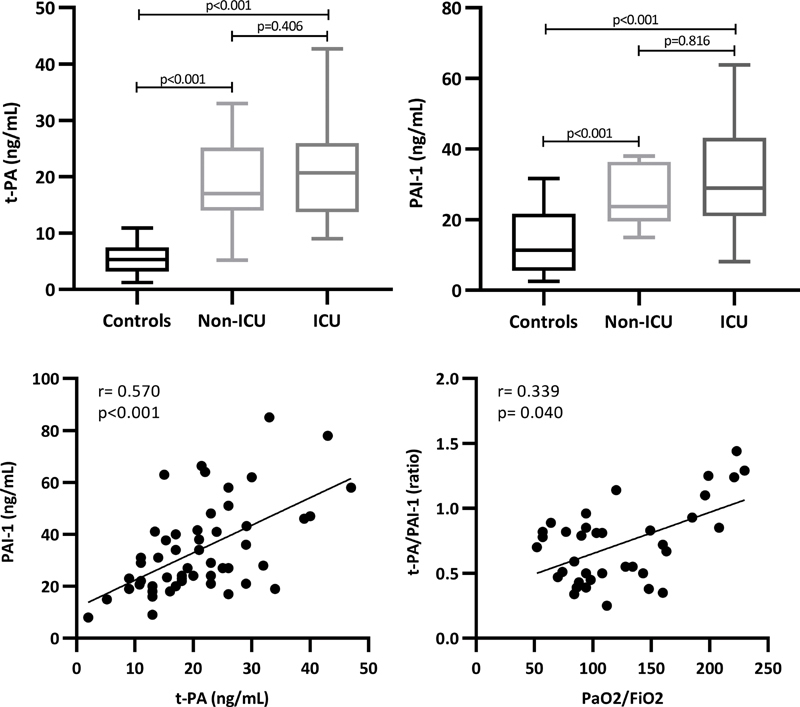
Fibrinolytic proteins levels (t-PA and PAI-1) in non-ICU and ICU patients compared with controls and their relationship with PaO2/FiO2 index.

As illustrated in Fig. 3 , both t-PA and PAI-1 plasma levels were significantly greater in patients compared with healthy controls, particularly in ICU patient group. Concentrations of these fibrinolytic proteins were significantly correlated to each other (r= 0.570; *p* < 0.001). Correlation analysis with other hemostatic variables, showed an inverse correlation between PAI-1 and D-dimer values (r= -0.326; *p* = 0.018), also by multivariable analysis corrected for age and gender (B= -0.277; *p* = 0.005). Among the clinical variables, a direct correlation was found between t-PA/PAI-1 ratio and PaO2/FiO2 index (r= 0.339, *p* = 0.040), also after adjustment for age and gender (B= 0.418; *p* = 0.013).

### Inflammatory Biomarker Investigation

**Fig. 4:**
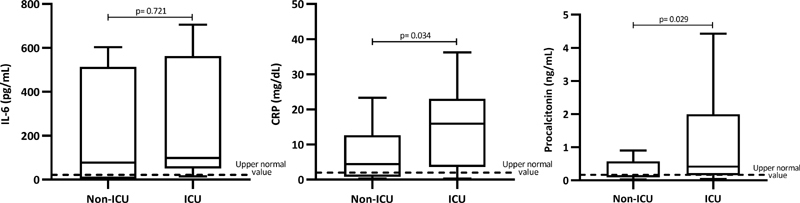
Inflammatory biomarkers in non-ICU and ICU patients.

**Fig. 5:**
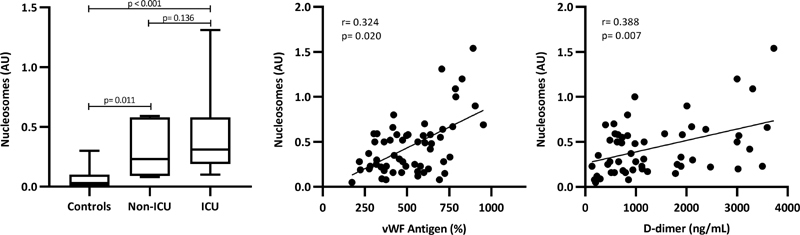
Nucleosomes levels in non-ICU and ICU patients compared with controls and their correlation with vWF antigen and D-dimer levels.

Fig. 4 shows the levels of inflammatory biomarkers in the overall cohort of patients and according to disease severity. Serum levels of IL-6 and CRP were above the upper normal reference value in 25% and 78% of patients, respectively, while procalcitonin values were >0.5 ng/mL in 62% of patients. Both CRP and procalcitonin were significantly higher in the ICU- compared with non-ICU patients. Serum procalcitonin concentrations (r= 0.649; *p* < 0.001) were significantly correlated with sTM values, whereas CRP levels correlated with plasma fibrinogen (r= 0.542, *p* < 0.001). Plasma levels of nucleosomes ( Fig. 4 ), as surrogate biomarkers for NETs, were found significantly increased in the overall group of patients, and especially in those hospitalized in ICU. Levels of this biomarker were found significantly ( *p* < 0.05) associated with vWF antigen (r= 0.324), FVIII (r= 0.433), sTM (r= 0.356), and D-Dimer (r= 0.388) ( Fig. 5 ), but not with leukocytes or neutrophil counts.

### Predictors of in Hospital Mortality

**Fig. 6:**
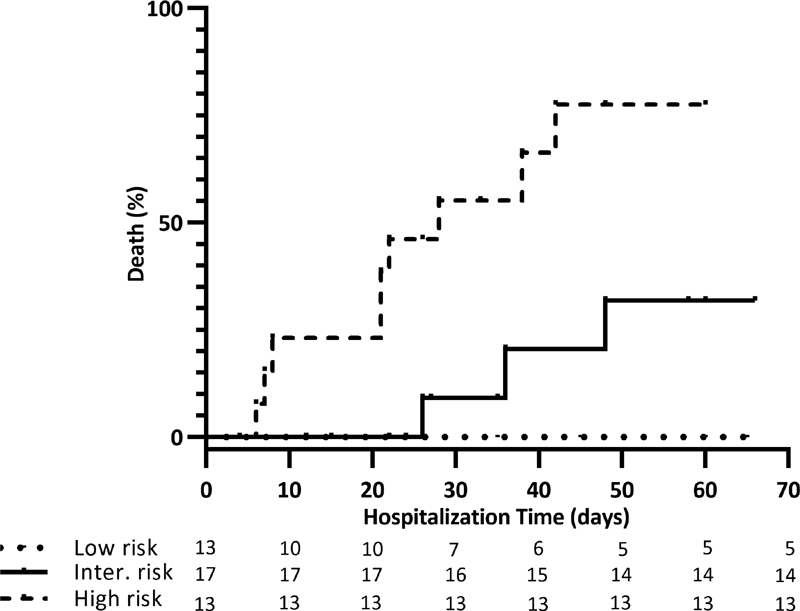
Cumulative incidence of death by the PaO2/FiO2 + vWF activity + Neutrophils score. The cumulative incidence of death was 0% in the low-risk (0/13; IC 95%, 0%), 32% in the intermediate-risk (3/17; IC 95%, 1.6–62%) and 78% in the high-risk group (9/13; IC 95%, 52–104%).

Patients were followed-up until hospital discharge or in-hospital death. After a median time of 30 days (range: 2 to 66 days) from hospitalization, death was documented in 13 patients (21% of total patients): 12 in the ICU and 1 in non-ICU settings. Compared with survivors, these patients presented at enrollment with lower levels of PaO2/FiO2 ( *p* = 0.009), elevated levels of vWF-antigen ( *p* = 0.036), vWF-activity ( *p* = 0.011), nucleosomes ( *p* = 0.011) and neutrophils ( *p* = 0.007) ( Table 3 ). After adjustment for age and gender by multivariable linear regression analysis, vWF-activity (B= 0.322, *p* = 0.031), neutrophil count (B = 0.325, *p* = 0.029), and PaO2/FiO2 (B= -0.355, *p* = 0.036) remained significantly associated with death ( Table 4 ). Furthermore, the predictive accuracy of the logistic regression model as assessed by the bootstrap-based optimism correction method, confirmed that the model was reliable and accurate ( Table 5 ). vWF activity, neutrophil count and PaO2/FiO2 were then utilized to generate a predictive score for mortality. To this aim, we first categorized the 3 variables as follow: categories for vWF levels and neutrophil counts were established on the basis of the 25th and 75th percentiles of variable's distribution in patients; while categories for PaO2/FiO2 were established according to the values used for defining ARDS severity 27 ( Table 6 ). Increasing points were assigned according to cut-off values, and a linear score was created by summing the points attributed to each variable category. According to this score, 3 different risk groups were defined: low (score ≤ 3), intermediate (score 4–6), and high (score ≥ 7). By ROC analysis, the score provided an AUC (area under the curve) of 0.823, with a sensibility of 63% and a specificity of 97%. The negative (NPV) and the positive predictive (PPV) values of the score were 84% and 91%, respectively. Fig. 6 describes the Kaplan-Meier survival curves of the 3 risk category groups: the cumulative incidence of death was 0% in the low-risk (0/13; IC 95%, 0%), 32% in the intermediate-risk (3/17; IC 95%, 1.6–62%) and 78% in the high-risk group (9/13; IC 95%, 52–104%).

**Table 3:** Biomarker comparison between non-survivors and survivors COVID-19 patients

	Non-survivors ( *n* = 13)	Survivors ( *n* = 50)	*p*
**vWF activity (%)**	490 (297–573)	406 (186–552)	**0.011**
**vWF antigen (%)**	627 (217–916)	474 (245–811)	**0.036**
** Neutrophils (10 ^9^ /L) **	14.6 (6.5–18.8)	7.6 (2.5–22.0)	**0.007**
**Nucleosomes (AU)**	0.64 (0.16–1.5)	0.25 (0.08–1.0)	**0.011**
**PaO2/FiO2 (%)**	86 (57–140)	128 (55–435)	**0.009**

Data are expressed as median and 5 ^th^ and 95 ^th^ percentiles. *p* is statistical significance by Mann-Whitney U test. vWF: von Willebrand factor.

**Table 4:** Influence of the predictive variables for mortality, after adjustment by age, gender, by multivariable linear regression analysis

	B coefficient	*P*
**Age**	0.117	**0.400**
**Male Gender**	0.078	**0.556**
** Neutrophils (10 ^9^ /L) **	0.345	**0.012**
**vWF activity (%)**	0.288	**0.039**
**PaO2/FiO2 (%)**	-0.301	**0.036**

Beta-coefficients from multivariable regression analysis assessing associations between predictive variables and mortality. P is statistical significance.

**Table 5:** Predictive accuracy of the logistic regression model of the score by the bootstrap-based optimism correction method

	Standard error	*P*
**Age**	0.005	**0.345**
**Male Gender**	0.138	**0.580**
** Neutrophils (10 ^9^ /L) **	0.013	**0.036**
**vWF activity (%)**	0.000	**0.020**
**PaO2/FiO2 (%)**	0.000	**0.021**

Bootstrap results based on 1,000 bootstrap samples. P is statistical significance.

**Table 6:** Risk score calculation for in-hospital mortality

vWF activity (%)	Points	
<150	**0**	
150–300	**1**	
>300–450	**2**	
>450	**3**	
** Neutrophils (10 ^9^ /L) **		
<6.7	**0**	
6.7–14	**1**	
>14	**2**	
**PaO2/FiO2 (mmHg)**		
>300	**0**	
200–300	**1**	
100–200 <100	**2** **3**	
**Risk score (points)**	**Risk category**	**Mortality risk according to score (%)**
≤ 3	**Low**	**0**
4–6	**Intermediate**	**32**
≥ 7	**High**	**78**

Abbreviations: ARDS, acute respiratory distress syndrome; vWF, von Willebrand factor.

## Discussion

Interaction between inflammation and the hemostatic system plays an important role in the onset of hypercoagulability, thrombosis, and mortality in severe COVID-19 patients. Studies suggest a dysregulated host immune response as the major cause of COVID-19-induced mortality; however a key role of the endothelium is likewise highlighted.

In our study, in a cohort of patients admitted for moderate to severe COVID-19 at the peak of the outbreak in Bergamo, we first performed an extensive investigation of different laboratory parameters of endothelial activation together with biomarkers of coagulation activation and inflammation in relation to disease severity and ARDS. The results of vWF measurement showed the presence of extremely elevated levels of this large multimeric protein of endothelial origin in the overall cohort of patients, mainly those of the ICU-patient group, with values beyond the upper normal range (i.e., > 150%) almost in all subjects. vWF antigen and activity increased in a similar manner and the levels of both were highly correlated. vWF is a circulating adhesive glycoprotein secreted by endothelial cells and platelets and its plasma levels have been found elevated in vasculitis, inflammation, aging, and diabetes, all conditions associated with endothelial dysfunction. Under normal blood flow conditions, vWF multimers exhibit low binding affinity for platelets, however, when exposed to increased hydrodynamic forces, they can efficiently bind to platelets. In addition, vWF contribute to blood coagulation as a carrier of coagulation FVIII. In our patients, measurement of FVIII activity showed significantly ( *p* < 0.001) higher levels of this procoagulant cofactor as compared with healthy controls, with the highest values in ICU group. FVIII is an acute phase reactant protein of hepatic origin which raises in the circulation under proinflammatory stimuli, similarly to fibrinogen, that was found increased as well in our patients and significantly correlated with CRP levels. Differently from FVIII, vWF is mainly synthetize by the endothelium, which in COVID-19 is activated not only by the inflammatory reaction to the infection but also by the direct viral attack. 31 32 33 It has been suggested that a pre-existing endothelial dysfunction combined with the direct assault of SARS-CoV-2 on vascular system may account for a high mortality of COVID-19 patients. However, in our study, we did not find any association between vWF levels and preexisting comorbidities or age. Endothelial perturbation in our subjects was also confirmed by the detection of significantly increased circulating levels of sTM. The rise in plasma levels of this endothelial protein is a frequent finding in patients with disorders associated with vascular perturbation, including infection, sepsis, and inflammation. 34 This phenomenon is related to the shedding of TM from the endothelial cell surface caused by pro-inflammatory stimuli and neutrophils derived proteases, 35 36 that impairs the endothelial anticoagulant PC pathway, thus favoring the generation of a procoagulant and pro-inflammatory local milieu within the pulmonary vasculature. 10 11 As well, in our study subjects, levels of sTM were significantly associated with concentrations of procalcitonin, a marker of ongoing infection.

Our results are coherent with those from previous studies describing the occurrence of endothelium activation in severe COVID-19, as shown by the detection of elevated plasma concentrations of vWF 6 25 and sTM. 5 37 Specifically, elevation of sTM was observed in 80% of COVID-patients with the highest values in critical patients and in those who did not survive. 5 Accordingly, in the present study, significantly higher sTM levels were detected in the moderate and severe ARDS group of patients as compared with those in the mild ARDS group.

Hemochromocytometric parameters showed significantly increased neutrophil and platelet counts and lymphopenia, characteristics that have been associated with disease severity and poor prognosis in COVID-19. 9 14 17 In this regard, extensive infiltration of pulmonary capillaries by activated neutrophil in autopsy specimens has been described as responsible of a worse outcome in COVID-19 patients. 38 39 Activated neutrophils release NETs in the extracellular space, as a main immune defense system against pathogens by a process named NETosis. Increased biomarkers of NETosis have been described in COVID-19 patients 16 40 41 42 and identified as predictors of respiratory failure and ARDS. We evaluated this defense mechanism by measuring nucleosomes as surrogate biomarkers of NETosis and found significantly increased levels in the overall group of patients, especially in those hospitalized in ICU, positively correlated with both biomarkers of endothelial dysfunction (i.e., vWF) and hypercoagulability (i.e., D-dimer).

Finally, in the present study we employed available clinical and laboratory features of patients to create an in-hospital mortality predictive risk assessment tool. After a median time of 30 days from hospitalization, 13 patients died. By multivariable linear regression analysis, we identified vWF activity, neutrophil count, and PaO2/FiO2 as biomarkers significantly associated with mortality. A linear predictive score for mortality was then created based on these variables that significantly discriminated patients in 3 different risk categories: low-risk with 0%, intermediate-risk with 32% and high-risk group with 78% mortality. Evidence for an association between increased circulating levels of biomarkers of endothelial damage and clinical outcomes have been previously reported. 12 25 43 However, in our study, for the first time, we could include vWF, a marker of endothelium perturbation, in a risk assessment model which significantly discriminates patients as regard to mortality.

To summarize, our study provides an extensive overview of the endothelial damage induced by SARSCoV-2 infection in hospitalized patients with virus-induced pneumonia and different degrees of disease severity. We found that the viral infection-induced endothelial abnormalities are exacerbated by the severity of the disease and strongly correlate with hypercoagulability and proinflammatory status, supporting the link between coagulation and inflammation. The combination of endothelial dysfunction with a generalized inflammatory state and innate immune system activation can very likely contribute to the overall hypercoagulable state of COVID-19 patients leading to thrombotic events, 16 44 and corroborates the strong association of hypoxia with endothelial damage. Furthermore, data provided by this study were useful for the generation of an in-hospital mortality risk score that, for the first time, links 3 important pathogenic pathways of severe COVID-19 disease: endothelial cells, neutrophils, and hypoxia.

The limitations of our study should be mentioned. First, our results are preliminary and hypothesis-generating, since the sample size is small and there is no external validation of the score. Next, our study was based on a single point measurement of the biomarkers, i.e., at study enrollment and a longitudinal biomarker study might provide further information including the temporal changes of biomarker levels and their possible relevance. The time from hospitalization to study entry ranged from 0 to 15 days (with a median time of 9 days), so that patients characteristics may be influenced by the hospitalization time. However, we did not find significant correlations between the levels of biomarkers and duration of hospital stay. Finally, our study was performed during the first wave of COVID-19 pandemic. Although it may be of interest to have recent data from second and even third waves, however, we believe that the conditions are not so different and our results are valid in all the phases of this pandemics, where pathogenesis is unchanged. In addition, this is the first report on hemostatic variables in the population of Bergamo, the first area in the word after China hit by SARS-CoV-2 infection, at the peak of pandemics.

Biomarkers of the present study were selected based on our experience and availability of the assays at the planning of the study. For exploring circulating endothelial activation/perturbation biomarkers we selected vWF and TM, together with PAI-1 and t-PA, which are well recognized endothelial biomarkers. It might be possible that the measurement of other endothelial markers, such as adhesion molecules and of TF/TFPI, might contribute to increase the value of the score.

To conclude, overall, our results support the basis for therapies aimed to stabilize the endothelium, such as anti-cytokine and anti-inflammatory agents, for reducing the mortality risk. This approach might be particularly important for susceptible subjects with a pre-exiting endothelial dysfunction, secondary to cardiovascular disease, hypertension, diabetes, obesity, and smoking, all conditions associated with unfavorable outcomes in COVID-19.

## Notes

**What is known on this Topic**

- Endothelial dysfunction is a common finding in COVID-19 patients, as highlighted by autoptic studies and the detection of increased levels of endothelial activation biomarkers.

**What does this Paper Add?**

- This study provides an extensive overview of the endothelial damage induced by SARS-CoV-2 infection in a prospective cohort of hospitalized COVID-19 patients.
- For the first time, vWF, a biomarker of endothelial dysfunction, together with neutrophil count and PaO2/FiO2 was included into a risk assessment model for prediction of in-hospital mortality.
- The study poses the basis of the use of vWF as a valuable biomarker for the rapid identification of subjects at higher risk of death
